# Behaviour of Zinc Complexes and Zinc Sulphide Nanoparticles Revealed by Using Screen Printed Electrodes and Spectrometry

**DOI:** 10.3390/s131114417

**Published:** 2013-10-25

**Authors:** Lukas Nejdl, Branislav Ruttkay-Nedecky, Jiří Kudr, Monika Kremplova, Natalia Cernei, Jan Prasek, Marie Konecna, Jaromir Hubalek, Ondrej Zitka, Jindrich Kynicky, Pavel Kopel, Rene Kizek, Vojtech Adam

**Affiliations:** 1 Department of Chemistry and Biochemistry, Faculty of Agronomy, Mendel University in Brno, Zemedelska 1, Brno CZ-613 00, Czech Republic; E-Mails: lukasnejdl@gmail.com (L.N.); brano.ruttkay@seznam.cz (B.-R.N.); george.kudr@centrum.cz (J.K.); monika.kempova@seznam.cz (M.K.); cernei.natalia3@gmail.com (N.C.); mariekon@centrum.cz (M.K.); zitkao@seznam.cz (O.Z.); 2 Central European Institute of Technology, Brno University of Technology, Technicka 3058/10, Brno CZ-616 00, Czech Republic; E-Mails: prasek@feec.vutbr.cz (J.P.); hubalek@feec.vutbr.cz (J.H.); paulko@centrum.cz (P.K.); kizek@sci.muni.cz (R.K.); 3 Department of Geology and Pedology, Faculty of Forestry and Wood Technology, Mendel University in Brno, Zemedelska 1, Brno CZ-613 00, Czech Republic; E-Mail: jindrak@email.cz; 4 Karel Englis College, Sujanovo Square 356/1, Brno CZ-602 00, Czech Republic

**Keywords:** electrochemical analysis, differential pulse voltammetry, screen printed electrode, spectrometry, Zn(II)

## Abstract

In this study, we focused on microfluidic electrochemical analysis of zinc complexes (Zn(phen)(his)Cl_2_, Zn(his)Cl_2_) and ZnS quantum dots (QDs) using printed electrodes. This method was chosen due to the simple (easy to use) instrumentation and variable setting of flows. Reduction signals of zinc under the strictly defined and controlled conditions (pH, temperature, flow rate, accumulation time and applied potential) were studied. We showed that the increasing concentration of the complexes (Zn(phen)(his)Cl_2_, Zn(his)Cl_2_) led to a decrease in the electrochemical signal and a significant shift of the potential to more positive values. The most likely explanation of this result is that zinc is strongly bound in the complex and its distribution on the electrode is very limited. Changing the pH from 3.5 to 5.5 resulted in a significant intensification of the Zn(II) reduction signal. The complexes were also characterized by UV/VIS spectrophotometry, chromatography, and ESI-QTOF mass spectrometry.

## 1. Introduction

Zinc is an essential element playing numerous crucial roles in organisms. It is involved especially in the synthesis of proteins and DNA [1], because zinc stabilizes the structure of chromatin and affects replication of DNA and transcription of RNA by regulating the activity of transcription factors for RNA and DNA polymerases [2]. Zinc is also essential to stabilize the structure of proteins containing zinc finger motifs [3]. Zinc is further closely connected with the production of insulin [4], and in light of this fact, zinc complexes could find an application in the treatment of diabetes [5]. Zn(II) complexes are able to modulate an inflammatory response by influencing the secretion and activity of several inflammation-related cytokines and enzymes [6]. Moreover, xylan-chitooligomer-zinc complex exhibited antioxidant and antimicrobial activity [7]. Transition metal complexes that are capable of cleaving DNA under physiological conditions are of interest in the development of anticancer drugs [8]. Cisplatin and related platinum-based drugs bind covalently to DNA, but they have side effects, especially, toxicity and acquired drug resistance, that requires the development of new drugs, which bind non-covalently to DNA, are less toxic and are target-specific. Among the non-platinum complexes for metal based chemotherapy, copper and zinc complexes have been much explored due to the fact that both copper and zinc are bioessential elements responsible for numerous bioactivities in living organisms [9–11]. Role of zinc and copper complexes as potential chemotherapeutic compounds have been confirmed, both complexes were able to bind and cleave DNA [12]. Zinc sulphide (ZnS) is one of the first semiconductors discovered and it has shown remarkable properties, versatility and a promise for novel diverse applications, including light-emitting diodes (LEDs), electroluminescence, flat panel displays, infrared windows, sensors, lasers, and biodevices, *etc.* [13]. Its atomic structure and chemical properties are comparable to more popular and widely known ZnO [14]. In the past decade, numerous results have been reported on the synthesis of nanometer scale semiconductor crystals (quantum dots, nanowires, nanorods, *etc.*) because their properties, due to quantum confinement effect, dramatically change and, in most cases, improve as compared with their bulk counterparts [15–17]. Among them, ZnS quantum dots (QDs) as semiconductor nanocrystals with a typical size of 2–10 nm have been attracting much interest [18]. An advantage of ZnS QDs is that they can be analysed electrochemically [19].

There is a wide range of well-established techniques for detection of metals, including the most widely used mass spectrometry and atomic absorption spectrometry. These methods are reliable and highly sensitive. On the other hand, they require expensive instrumentation and involve time-consuming procedures. Electrochemical methods represent another class of widely used techniques for the detection of metal ions. Anodic stripping voltammetry has become one of the most important techniques [20–22] in this field, together with hanging mercury drop electrode (HMDE) [23–25]. The disadvantage of this method is the difficulty of miniaturization, especially due to the hanging drop, which needs the supply of gas. Another disadvantage of the mercury electrode is its limited modification possibilities, a small anodic range (limited by the oxidation of mercury) and the high toxicity of mercury. Mercury electrodes also cannot be used in a flow system.

Despite their sensitivity issues, screen printed electrodes (SPEs) are a suitable alternative to HMDE. The low acquisition costs of lithographic equipment have enabled the widespread use of disposable SPEs as biosensors and chemical sensors in microfluidic systems. Microfluidics is a technology that requires lower volumes of sample, increases the speed of analysis and response time, allowing a massive parallelization for high-throughput analysis, and reducing the cost of fabrication of biosensors [26–28]. In recent years, methods involving the coupling of microfluidics with electrochemical techniques have been increasing because of the benefits associated with miniaturization, automation, sensitivity and specificity [29–35].

Based on the abovementioned facts we investigated the combination of zinc as a central atom, 1,10-phenanthroline (phen) as a versatile N-N chelating aromatic ligand that can interact with DNA by π-π interaction and histidine as an amino acid with a side chain aromatic ring. Aromatic ligands also play an important role in enhancing DNA binding and cleavage activity. We also selected histidine as a ligand because it is known that amino acids/peptides recognize a specific base sequence of DNA and that aromatic ring contributes to the stabilization of proteins through hydrophobic interactions and the formation of hydrophilic environments [36].

## 2. Experimental Section

### 2.1. Chemicals and Materials

ZnCl_2_, Zn(NO_3_)_2_·6H_2_O, L-histidine, 1,10-phenanthroline, 3-mercaptopropionic acid, and Na_2_S·9H_2_O, all of ACS purity, were purchased from Sigma-Aldrich (St. Louis, MO, USA). Stock solutions were prepared using ACS water immediately before use. pH values were measured using an inoLab Level 3 instrument (Wissenschaftlich-TechnischeWerkstatten GmbH; Weilheim, Germany). Deionised water underwent demineralization by reverse osmosis using an Aqua Osmotic 02 system (Aqua Osmotic, Tisnov, Czech Republic) and was subsequently purified using a Millipore RG system MiliQ water, 18 MΩ, (Millipore Corp., Billerica, MA, USA).

#### 2.1.1. Preparation of Zinc Nitrate Hexahydrate

Stock solution of zinc nitrate (1 mM) was prepared by dissolving of zinc nitrate hexahydrate (0.297 g) in water (1 L).

#### 2.1.2. Preparation of Zn(phen)(his)Cl_2_

ZnCl_2_ (0.136 g) was dissolved in water (10 mL). A suspension of histidine (0.155 g) and 1,10-phenanthroline (0.2 g) in water (90 mL) was added to the ZnCl_2_ solution under constant stirring. The reaction mixture was placed in an ultrasonic bath for 30 min and dissolution of reaction components occurred. After that, the reaction mixture was stirred overnight. The resulting colourless solution was used for measurements.

#### 2.1.3. Preparation of Zn(his)Cl_2_

Preparation of the complex was the same as for Zn(phen)(his)Cl_2_, but only histidine was added to the ZnCl_2_ solution. A colourless solution was obtained.

#### 2.1.4. Preparation of ZnS Quantum Dots (QDs)

ZnS MPA (MPA = 3-mercaptopropionic acid) QDs were prepared using the slightly modified method published in [18,19,37]. Zinc nitrate hexahydrate Zn(NO_3_)_2_·6H_2_O (0.03 g, 0.1 mM) was dissolved in ACS water (25 mL). 3-Mercaptopropionic acid (35 μL, 0.4 mM) was added slowly to the stirring solution. Afterwards, the pH was adjusted to 9.1 with 1 M NH_3_ (1.5 mL). Sodium sulphide nonahydrate Na_2_S·9H_2_O (0.024 g, 0.1 mM) in ACS water (22 mL) was poured into the first solution under vigorous stirring. The obtained colourless solution was then stirred for 1 h.

### 2.2. UV/VIS —Spectrophotometry

Absorption spectra were recorded using a SPECORD 210 spectrophotometer (Analytik Jena, Jena, Germany) in the range 200–400 nm and in steps of 1 nm. Quartz cuvettes with 1 cm optical path (Hellma, Essex, UK) were used. The cell with cuvette was thermostated to 20 °C with a Julabo thermostat (Labortechnik, Wasserburg, Germany). Absorption spectra were recorded after 60 min of interaction and were evaluated using the WinASPECT program, version 2.2.7.0.

#### Spectral Analysis of Zinc

Zinc forms a red chelate complex with 2-(5-bromo-2-pyridylazo)-5-(N-propyl-N-sulfo-propylamino) phenol (Nitro-PAPS) with an absorption maximum at λ = 560 nm. The colour intensity is proportional to the total zinc concentration in the sample. A volume of 800 μL of reagent (Greiner, Frickenhausen, Germany), 0.02 mM 2-(5-bromo-2-pyridylazo)-5-(N-propyl-N-sulphopropylamino)phenol, 170 mM sodium citrate, and 4 mM dimethylglyoxime in 200 mM bicarbonate buffer, pH 9.8) was pipetted into plastic cuvette and subsequently 40 μL of the sample was added. Spectra were recorded after 5 min long incubation of a reagent with a sample. After a measurement, cuvettes were rinsed with deionised water and dried with nitrogen.

### 2.3. Screen Printed Electrodes' System

#### 2.3.1. Electrode System Design

The electrode system was designed and fabricated as a disposable planar three-electrode sensor in the LabSensNano laboratories (Brno University of Technology, Brno, Czech Republic). The 25.4 × 7.2 mm dimensions of the 0.625 mm thick alumina electrode substrate were achieved by a dividing of standard 2 × 2″ piece of the substrate. The shape and theoretical working area of electrodes were designed according to the previous optimization process [38]. In accordance with the optimization results, the working electrode (WE) was designed to be as large as possible (in this case geometrically comparable with the diameter of the GCE of 3 mm with a working area of 7.1 mm^2^), reference electrode (RE) 1.3 mm^2^ and auxiliary electrode (AE) 6.2 mm^2^.

#### 2.3.2. Electrode System Fabrication

The screen-printed sensor was fabricated using a semiautomatic Aurel C880 screen-printer (Aurel Automation, Modigliana, Italy) and fired using a BTU fast fire furnace for the thick-film processing (BTU, North Billerica, MA, USA). The conductive layer was fabricated from AgPdPt-based paste (ESL 9562-G). The protective layer was fabricated from dielectric paste (ESL 4917). AE was fabricated from Pt-based paste (ESL 5545). All cermet pastes were obtained from ESL ElectroScience Europe, Berkshire, UK and fired at 850 °C according to the recommended values in the products datasheets. WE was a screen-printed electrode fabricated using a special carbon paste for electrochemical sensor electrodes (DuPont BQ221, DuPont Company, Wilmington, DE, USA) and cured at 130 °C for 10 min according to the manufacturer's datasheet. RE was screen-printed using a special polymer Ag/AgCl paste (DuPont 5874, Ag:AgCl ratio 65:35) and dried at 120 °C for 5 min.

#### 2.3.3. Microfluidic Analysis with Electrochemical (Differential Pulse Voltammetry) Detection

Printed electrodes (three-electrode system) and a flow cell were used. The flow cell was designed in the shape of a cuboid with sides of 1 cm (width) × 1.5 cm (height) × 3 cm (length). The reaction zone was designed for 10 μL of analyte with 0.7 mm wide inlet and outlet channel. The sample was injected using a peristaltic pump (Amersham Biosciences, Glattbrugg, Sweden). Changes in the reduction signal were recorded with a PGSTAT 101 potentiostat (Metrohm, Herisau, Switzerland) and the results were evaluated by the NOVA 1.8 software (Metrohm). Settings of the potentiostat were as it follows: initial potential −1.7 V, end potential −0.1 V, step potential 0.01 V, modulation amplitude 0.1 V, modulation time 0.004 s, interval time 0.1 s, equilibration time 5–130 s. Samples were diluted with acetate buffer pH 3.3–5.5 and measured at temperature from 10 to 50 °C (both parameters were optimized). Screen-printed sensors and analysis solutions were thermostated (10–50 °C) before measurement by a Julabo thermostat.

### 2.4. Differential Pulse Voltammetric Determination of Zinc Using HMDE

Determination of zinc by the differential pulse voltammetry was performed with a 663 VA Stand (Metrohm) using a standard cell with three electrodes. A hanging mercury drop electrode (HMDE) with a drop area of 0.4 mm^2^ was the working electrode. An Ag/AgCl/3M KCl electrode was the reference and a glassy carbon electrode was the auxiliary. Analysed samples were deoxygenated prior to measurement by purging with argon (99.999%). Acetate buffer (0.2 M CH_3_COONa + CH_3_COOH, pH 5), which was exchanged after each analysis, was used as a supporting electrolyte. The parameters of the measurement were as follows: initial potential of −1.3 V, end potential −0.6 V, deoxygenating with argon 90 s, accumulation time 240 s, time interval 0.04 s, step potential 5 mV, modulation amplitude 25 mV, deposition potential −1.15 V, volume of injected sample: 20 μL, volume of measurement cell 2,000 μL (20 μL of sample + 1,980 μL acetate buffer).

### 2.5. Atomic Absorption Spectrometry (AAS)

Determination of zinc was carried out on a 240FS atomic absorption spectrometer (Agilent Technologies, Santa Clara, CA, USA) equipped with flame atomization. The zinc hollow cathode lamp (Agilent) operated at the current of 5 mA. Zinc was measured on the primary wavelength of 213.9 nm with a spectral bandwidth of 1.0 nm. A mixture of air and acetylene was used to atomize the zinc in the flame. Deuterium correction of background was used and the signal was measured in an integration mode for 2 s.

### 2.6. Amino Acid Analysis (AAA)

An AAA 400 liquid chromatography apparatus (Ingos, Prague, Czech Republic) was used to determine the amino acid histidine. The system consisted of a glassy filling chromatographic column and a steel precolumn, two chromatographic pumps for transportation of elution buffers and derivatization reagent, a cooled carousel for 25 test tubes of 1.5–2.0 mL volume, a dosing valve, a heat reactor, a visible detector and a cooled chamber for the derivatization reagent. The glassy chromatographic column (i.d. 3.7 mm and 350 mm length) was filled with LG ANB strong catex in sodium cycle (Spolchemie, Usti nad Labem, Czech Republic) with particles of an average size of 12 μm and a netting of 8%. The glassy column was tempered by a thermostat working in the interval from 35 to 95 °C range. The precolumn was filled with LG KS0804 ionex (Ingos). Chromatographic columns are able to work at a flow rate 0.01–10 mL/min under a maximum pressure of 40 MPa. Volume of injected sample was 100 μL with an accuracy of application RSD of about 1%. A two-channel VIS detector with a 5 μL flow volume cuvette was operated at wavelengths of 440 and 570 nm. Ninhydrin solution (Ingos) was used as a derivatization reagent. Ninhydrin was dissolved in a solution containing 75% (*v*/*v*) of the organic solvent methyl cellosolve (Ingos) and 25% (*v*/*v*) of 4 M acetate buffer (pH 5.5). SnCl_2_ (Lachema, Brno, Czech Republic) was used as a reducing agent. Derivatization reagent was stored for the whole time under an inert atmosphere (N_2_) with cooling at 4 °C. During the analysis, the flow rate of mobile phase was set at 0.3 mL/min under a pressure ranging from 4.5 to 6.0 MPa. Reactor temperature was set to 120 °C.

### 2.7. Electrospray Ionization Quadrupole Time-of-Flight (ESI-QTOF) Mass Spectrometer

Samples was prepared in acetonitrile (MS purity) with a 1% addition of formic acid and characterized by a Bruker Maxis Impact Q-TOF mass spectrometer (Bruker, Billerica, MA, USA). ESI source was operated in the positive mode. Voltage of an electrospray capillary was set to 3,500 V with a flow rate of nebulizing gas of 4 L/min and a drying gas temperature was set to 350 °C. Flow rate of samples was set up to 180 μL/h.

### 2.8. Scanning Electron Microscopy (SEM) Characterisation of Modified Dowexmicroparticles

Structural and elemental composition of the screen-printed sensor were characterised by an electron microscope. For documentation of the selected nanomaterials, a FEG-SEM MIRA XMU instrument (Tescan, a.s., Brno, Czech Republic) was used. This model is equipped with a high brightness Schottky field emitter for low noise imaging at fast scanning rates. The SEM was fitted with Everhart-Thronley type of SE detector, a high speed YAG scintillator based BSE detector, a panchromatic CL Detector and EDX spectrometer. The MIRA 3 XMU system is based on a large specimen chamber with motorized stage movements of 130 × 130 mm. Samples were coated by 10 nm of carbon to prevent charging. A carbon coater K950X (Quorum Technologies, Grinstead, UK) was used. For automated acquisition of selected areas, a TESCAN proprietary software tool called Image Snapper was used. The software enables automatic acquisition of selected areas with defined resolution. Different conditions were used in order to obtain either minimum analysis time or maximum detail during overnight automated analysis. An accelerating voltage of 15 kV and beam currents of about 1 nA gave satisfactory results regarding maximum throughput.

## 3. Results and Discussion

**Figure 1.**
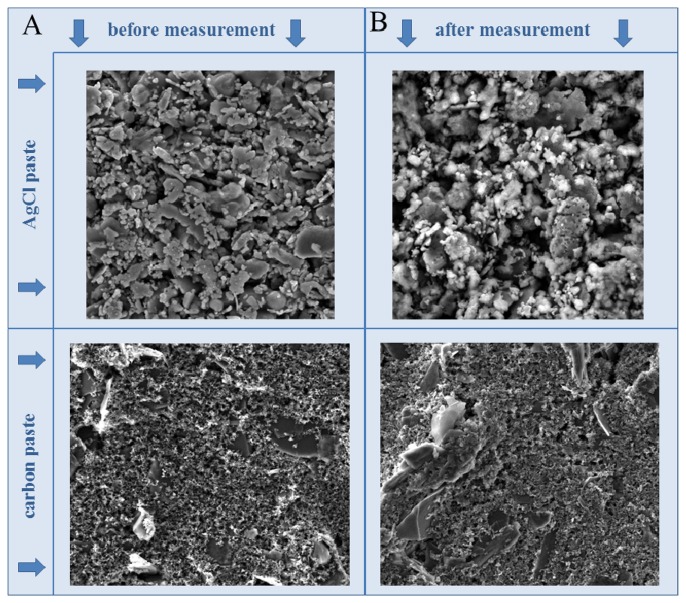
SEM photos of AgCl and carbon electrodes of SPE before and after measurement.

In this study, we focused on the electrochemical analysis of four zinc compounds using a microfluidic system that consisted of a flow cell for printed sensors (electrodes). The surface of the printed electrodes, consisting of carbon and silver chloride (AgCl) paste, was characterized using SEM (Figure 1). It clearly follows from the results obtained that the surface of the working electrode did not change during the measurement, however, the surface of the reference one was slightly changed. This is given by chloride included in the paste which is little bit dissolved into the solution during the measurement. This could be associated with the fact that the electrochemical reaction monitored on the surface of the working electrode influenced the reference one. This is one of the reasons of the disposability of the printed electrodes. The great advantage of carbon paste is its ability to be modified easily by various agents (nanoparticles) to achieve better sensitivity and selectivity [39–42].

In recent years, a growing interest in fast, reliable and inexpensive sensors to determine the different types of analytes in biomedical, environmental and industrial samples has been shown [43–45]. The method described in the Section 2.3 (*Three electrode system*) was chosen because of easy instrumentation (easy to use) and a possibility of setting variable flows [46].

**Figure 2.**
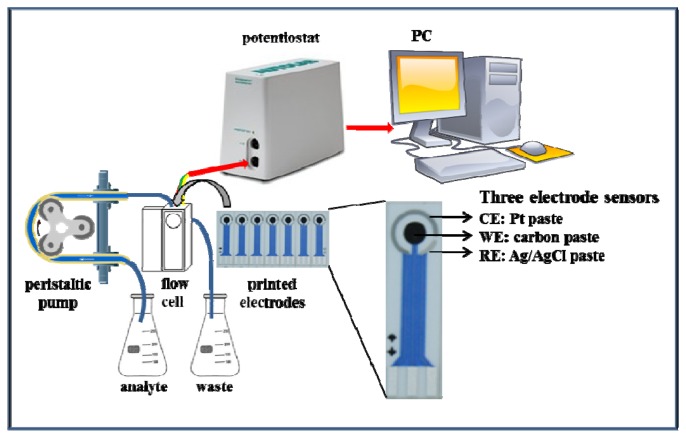
Schematic diagram of the microfluidic system with electrochemical detection.

The functionality of the suggested microfluidic system with electrochemical detection was tested firstly on zinc nitrate. The microfluidic system consisted of a workstation (PC), a potentiostat, a peristaltic pump and a flow cell for printed electrodes (Figure 2). The great advantage of this system is easy operation and service, thus, such systems can be used in a wide range of applications [47–49].

**Figure 3.**
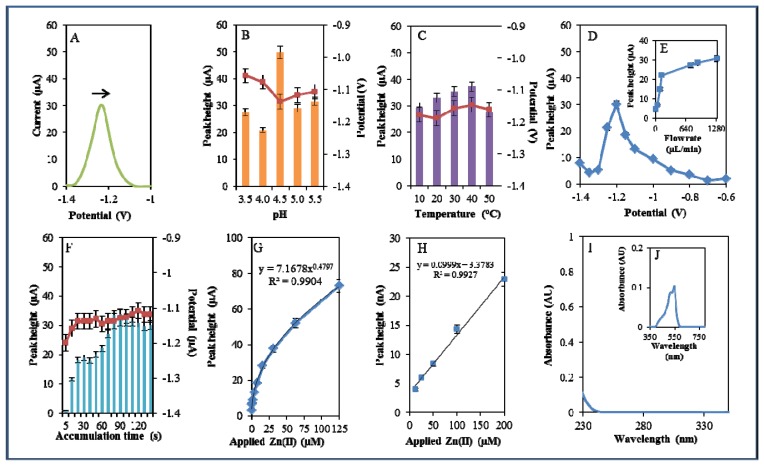
Electrochemical and spectrophotometric analysis of Zn(NO_3_)_2_. Basic parameters were 20 μM Zn(NO_3_)_2_ in 0.2 M acetate buffer, pH 5, temperature 20 °C unless stated otherwise. Electrochemical analysis of the Zn (II) ions using DPV (**A**) DP voltammogram of zinc nitrate. The effect of (**B**) pH (3.5–5.5), (**C**) temperature (10–50 °C), (**D**) applied potential (−1.4–−0.6 V), (**E**) applied flow rate (0–1,280 μL/min), (**F**) time of accumulation (5–120 s), and (**G**) Calibration curve of Zn (II) ions using DPV, dependence of used concentration of zinc nitrate (0–125 μM) on the change of reduction signal. (**H**) Dependence of used concentration of zinc nitrate on the change of reduction signal monitored using HMDE. In Figures **B**, **C**, and **F**, the blue columns represent height of signals (peaks, left scale), red squares indicate the potential at which the height of signals was detected (right scale). Spectrophotometric analysis of the Zn (II) ions. (**I**) Spectrophotometric record of the Zn(II) ions within the range from 230 to 380 nm. (**J**) Spectrophotometric record of the coloured product of the reaction of Zn(NO_3_)_2_ with 5-brom-PAPS with absorption maximum at λ = 550 nm.

During electrochemical analysis of 20 μM zinc nitrate reduction the zinc signals were monitored [50] under strictly defined conditions (pH, temperature, flow rate, time accumulation, applied potential) and are shown in Figure 3A. Firstly, we monitored the dependence of the reduction signal of Zn(II) ions on the change in pH value. Acetate buffers (pH 3.5, 4, 4.5, 5 and 5.5) were used for all electrochemical analyses. Application of 0.2 M acetate buffer, pH = 4.5, led to a twofold increase of intensity of the signal (Figure 3B). Temperature (10–50 °C), potential (from −0.6 to −1.4 V), flow rate (from 0 to 1,280 μL/min), and time of accumulation (from 5 to 140 s) were other tested parameters. The best response (signal intensity) of the Zn(II) reduction signal was achieved at 40 °C (Figure 3C), applied potential −1.2 V (Figure 3D), flow rate 1,280 μL/min, where the most significant changes were evident up to the flow rate 133.5 μL/min (Figure 3E), and accumulation time 90 s (Figure 3F). With increasing time of accumulation signal intensity first increased (up to 90 s) and then slightly decreased (90–120 s, Figure 3F). The potential was significantly shifted to more positive values with the increasing time of accumulation.

Calibration curve of Zn(II) ions using differential pulse voltammetry (DPV) is shown in Figure 3G. The dependence of concentration on the change in electrochemical signal was exponential with a coefficient of determination R^2^ = 0.9909 (Figure 3G). To compare our electrochemical results, we also utilized standard hanging mercury drop electrode (HMDE) for determination of zinc(II) ions. The measured dependence of concentration on the change of signal was linear with a coefficient of determination R^2^ = 0.9927 and is shown in Figure 3H. It clearly follows from the results that the results obtained using SPE are comparable to those obtained by HMDE. Zinc nitrate was further characterized by UV/VIS spectrophotometry. The absorption spectrum was monitored within the range from 230 to 350 nm. In this range, no absorption maxima were detected, and the signal was only slightly increased at λ = 230 nm (Figure 3I). 2-(5-Bromo-2-pyridylazo)-5-[N-propyl-N-(3-sulfopropyl) amino]phenol disodium salt dehydrate (5-brom-PAPS) was used to detect Zn. This compound creates a coloured product with Zn(II) that can be detected at λ = 550 nm [51]. The spectrophotometric record of the product with absorption maximum is shown in Figure 3J. In the next step, we characterized electrochemical behaviour of two zinc complexes, (Zn(phen)(his)Cl_2_ andZn(his)Cl_2_), and zinc-based nanoparticles (ZnS QDs).

### 3.1. Microfluidic Analysis with Electrochemical Detection of Zn(phen)(his)Cl_2_ Complex

**Figure 4.**
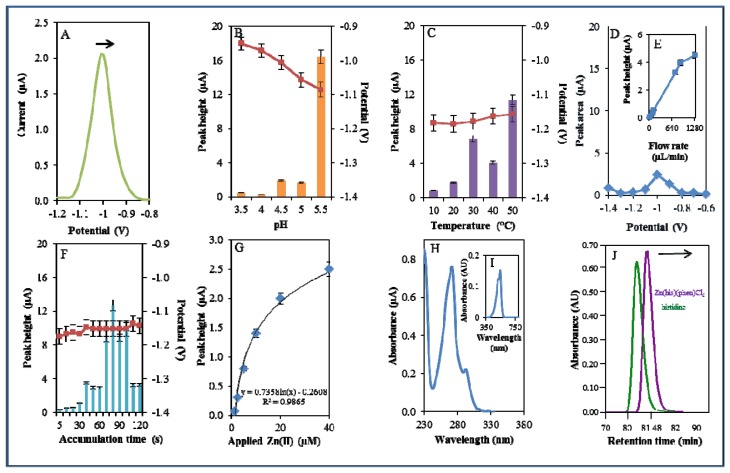
Electrochemical, spectrophotometric and chromatographic analysis of Zn(phen)(his)Cl_2_ complex. Basic parameters were 20 μM Zn(phen)(his)Cl_2_ in 0.2 M acetate buffer, pH 5, temperature 20 °C unless stated otherwise. Electrochemical analysis of the Zn(phen)(his)Cl_2_ complex using DPV. **A**) DP voltammogram of Zn(phen)(his)Cl_2_. Change in the height of reduction signal of Zn(phen)(his)Cl_2_ influenced by (**B**) pH (3.5-5.5), (**C**) temperature (10–50 °C), (**D**) applied potential (−1.4–−0.6 V), (**E**) applied flow rate (0-1,280 μL/min), (**F**) time of accumulation (5–120 s), and (**G**) Calibration curve of Zn (II) ions using DPV, dependence of used concentration of Zn(II) ions in the complex (1–40 μM) on the change of reduction signal. In Figures **B**, **C**, and **F**, the blue columns represent height of signals (peaks, left scale), red squares indicate the potential at which the height of signals was detected (right scale). Spectrophotometric and chromatographic analysis of the Zn(phen)(his)Cl_2_ complex. (**H**) Spectrophotometric record of Zn(phen)(his)Cl_2_ measured within the range from 230 to 380 nm. (**I**) Detection of Zn(II) ions using spectrophotometric record of the colored product of the reaction of Zn(phen)(his)Cl_2_ with 5-brom-PAPS with absorption maximum recorded at 550 nm. (**J**) Chromatogram demonstrating dependence of absorbance of histidine and Zn(phen)(his)Cl_2_ on retention time.

A microfluidic system with electrochemical detection was used to study the Zn(phen)(his)Cl_2_ complex. In the experiment, we monitored the reduction signal of 20 μM Zn(phen)(his)Cl_2_ (Figure 4A) under strictly defined experimental conditions as in the case of zinc nitrate. The highest signal was observed at pH 5.5. The recorded signal was on average eight times higher in comparison with lower pH values (Figure 4B). The change from acidic (pH 3.5) to less acidic (pH 5.5) pH values was accompanied by a significant shift of the potential to more negative values. Bound Zn(II) in the complex is released by an increase in pH value. This fact has been confirmed in experiments with the changing pH (Figure 4B). A similar release of enclosed compounds depending on a change in pH value has been shown in the case of apoferritin [52,53] or in dual cargo delivery from mesoporous silica nanoparticles with a metal-latched nanogate [54].

An increase in temperature (10–50 °C) had a contrary effect, and the signal was shifted to more positive values and the highest signal occurred at 50 °C at a potential of −1.15 V (Figure 4C). Changes in potential were further monitored amperometrically within the range from −1.4 to −0.6 V. The highest signal was recorded at a potential of −1 V (Figure 4D). The best response of the signal was well evident at the flow rate 640 μL/min (Figure 4E). The most suitable time of accumulation was established as 80 s. The potential showed a moderate shift to more positive values with the increasing time of accumulation (Figure 4F). Calibration curve of Zn(II) ions using DPV is shown in Figure 4G. The dependence of concentration on the change in electrochemical signal was logarithmic in concentration range 1–40 μM (Figure 4G)

The complex was further investigated by UV/VIS spectrophotometry. The absorption spectrum was monitored within the range from 230 to 350 nm. The complex showed two absorption maxima (λ = 270 and 291 nm, Figure 4H). Complex was further mixed with 2-(5-bromo-2-pyridylazo)-5-[N-propyl-N-(3-sulfopropyl)amino]phenol disodium salt dihydrate and incubated similarly as in the previous case. Formed colour product was identified at the same wavelength (λ = 550 nm) as zinc nitrate, which proved the presence of zinc [51]. Because the investigated complex contains the amino acid histidine, it was investigated using an automated amino acids analyser. L-Histidine (his) is an essential amino acid involved in the chelation of metal ions [55,56]. The chromatogram of the complex was compared with the chromatogram of a histidine standard. It was shown that signal of the standard occurred at a shorter retention time (80.67 min) than the signal of the complex (81.56 min). This fact indicates creation of the complex (Figure 4J).

### 3.2. Characterization of Zn(phen)(his)Cl_2_ by ESI-QTOF Mass Spectrometry

**Figure 5.**
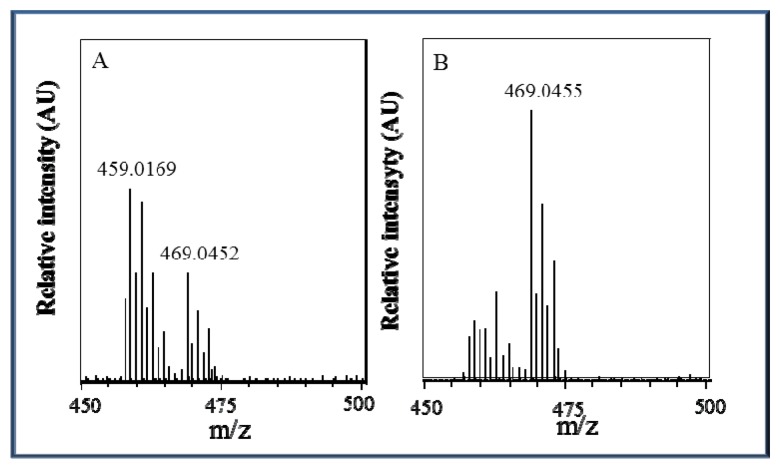
ESI-QTOF mass spectrum of 10 μM Zn(phen)(his)Cl_2_ measured within the range from 450 to 500 m/z in 0.2 M in the presence of acetate buffer (**A**) pH 3.5 and (**B**) pH 5.5.

Due to the behaviour of the zinc complex under various pH conditions and the non-linear calibration dependence, we attempted to analyse this complex using mass spectrometry. The ESI+ mass spectra display an intense signal at *m*/*z* = 469 that corresponds to the molecular ion Zn(his)(phen)Cl_2_H^+^ and a signal at *m*/*z* = 459 that corresponds to the molecular ion Zn(his)(phen)(ACN)(H_2_O)H^+^ with solvent molecules coordinating to the zinc central ion replacing coordinated chlorine ions. The height (intensity) of these peaks is closely connected with changes in pH value, as it can be seen in Figure 5A,B, respectively. At pH 5.5, the Zn(his)(phen)Cl_2_ complex is more stable than at pH 3.5 and coordination of chlorine ions is preferred. In the spectra, there are also signals with lower *m/z* = 383, 367, and 314 that correspond to different complex fragments. All *m*/*z* interpretations are based on ^35^Cl and ^64^Zn, respectively.

### 3.3. Microfluidic Analysis with Electrochemical Detection of Zn(his)Cl_2_ Complex

**Figure 6.**
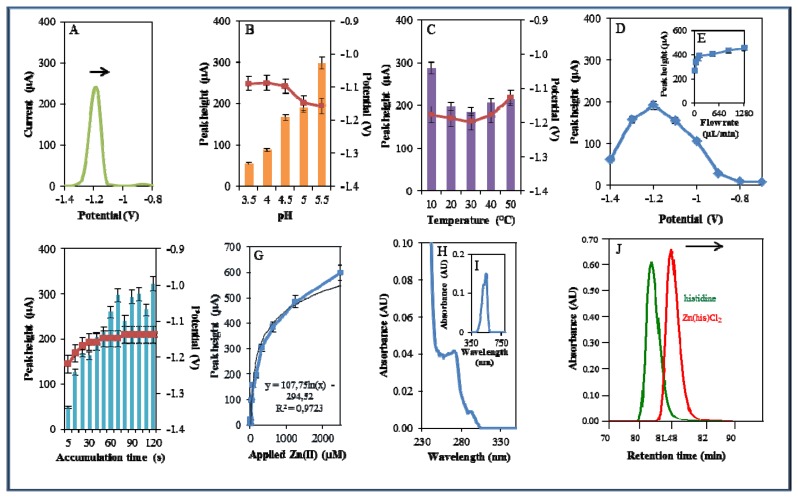
Electrochemical, spectrophotometric and chromatographic analysis of Zn(his)Cl_2_ complex. Basic parameters were 200 μM Zn(phen)(his)Cl_2_ in 0.2 M acetate buffer, pH 5, temperature 20 °C unless stated otherwise. Electrochemical analysis of the Zn(his)Cl_2_ complex using differential pulse voltammetry (DPV). (**A**) DP Zn(his)Cl_2_ voltammogram. The effect of (**B**) pH (3.5–5.5), (**C**) temperature (10–50 °C), (**D**) applied potential (−1.4–−0.6 V), (**E**) applied flow rate (0–1,280 μL/min), (**F**) time of accumulation (5–120 s), and (**G**) Calibration curve of Zn (II) ions using DPV, dependence of used concentration of Zn(II) ions in the complex (1–2,500 μM) on the change of reduction signal. In Figures **B**, **C**, and **F**, the blue columns represent height of signals (peaks, left scale), red squares indicate the potential at which the height of signals was detected (right scale). Spectrophotometric and chromatographic analysis of the Zn(his)Cl_2_ complex. (**H**) Spectrophotometric record of complex measured within the range from 230 to 380 nm. (**I**) Detection of Zn(II) ions using spectrophotometric record of the colored product of the reaction of Zn(his)Cl_2_ with 5-brom-PAPS with absorption maximum recorded at 550 nm. (**J**) Chromatogram demonstrating dependence of absorbance of histidine and Zn(his)Cl_2_ complex on retention time.

The other studied zinc complex did not contain a phenanthroline nitrogen-containing ligand, but only the amino acid histidine. Metal complexes of histidine may exhibit catalytic activity, especially with molybdenum, in oxidation reactions, as was shown by Vassilev *et al.* [55]. Due to the catalytic properties of Zn-his complexes, their possible application as catalysts was investigated [56]. In this study, Zn(his)Cl_2_was studied by the same way as Zn(phen)(his)Cl_2_ (Figure 6A). The highest signal was determined using 0.2 M acetate buffer of pH 5.5. This signal was six times higher compared to that measured in the presence of 0.2 M acetate buffer pH 3.5. The movement from (pH 3.5) to less acidic (pH 5.5) pH was accompanied by the shift of the potential to more negative values (Figure 6B) as in the case of Zn(phen)(his)Cl_2_.

The increasing temperature (20–50 °C) had almost no effect on the signal height. Higher temperature (30–50 °C) led to a signal shift to more positive values. Temperature of 10 °C and applied potential −1.18 were determined as the most advantageous (Figure 6C). Changes in potential were further monitored amperometrically within the range from −1.4 to −0.6 V. The highest signal was observed at potential −1.2 V (Figure 6D) similarly to that observed for zinc nitrate. Optimal signal was determined at the flow rate 320 μL/min (Figure 6E). The most suitable time of accumulation was determined as 120 s, where the potential showed a shift to more positive values with increasing time (potential shifted in the range of the time of accumulation 5–30 s, after that it was constant, Figure 6F). Calibration curve of Zn(II) ions using DPV is shown in Figure 6G. The dependence of concentration on the change in electrochemical signal was logarithmic with a coefficient of determination R^2^ = 0.9723 (Figure 6G). Zn(his)Cl_2_ was finally characterized by UV/VIS spectrophotometry. The absorption maximum was recorded at λ = 270 nm. Presence of Zn(II) in 500 μM Zn(his)Cl_2_ was proved spectrophotometrically by the colour reaction recorded at λ = 550 nm [51]. Complex was also investigated by automated analyser of amino acids by the same way as Zn(phen)(his)Cl_2_. Signal of the standard occurred at a shorter retention time (80.67 min) compared to that of studied complex (81.18 min). This fact verified creation of complex (Figure 6J). Studied complex (Zn(his)Cl_2_) eluted 0.38 min earlier than that of Zn(phen)(his)Cl_2_. This result verified differences between these two studied complexes.

### 3.4. Flow Analysis of ZnS QDs

**Figure 7.**
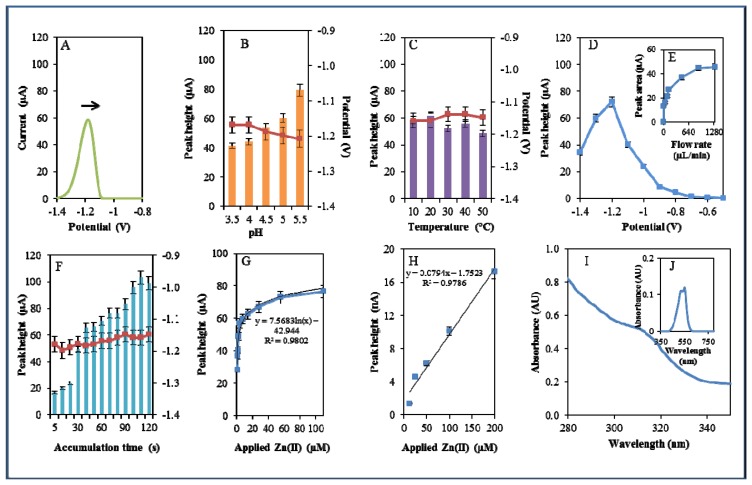
Electrochemical and spectrophotometric analysis of ZnS QDs. Basic parameters were 20 μM ZnS QDsin 0.2 M acetate buffer, pH 5, temperature 20 °C unless stated otherwise. Electrochemical analysis of the ZnS QDs using differential pulse voltammetry (DPV). (**A**) DP voltammogram of ZnS QDs. The effect of (**B**) pH (3.5–5.5; 0.2 M acetate buffer), (**C**) temperature (10–50 °C), (**D**) applied potential (−1.4–−0.6 V), (**E**) applied flow rate (0–1,280 μL/min), (**F**) time of accumulation (5–120 s), and (**G**) Calibration curve of Zn(II) ions using DPV, dependence of used concentration of Zn(II) ions in ZnS QDs (1–110 μM) on the change of reduction signal. (**H**) Dependence of applied concentration of ZnS QDs on the change of signal recorded using HMDE. In Figures **B**, **C**, and **F**, the blue columns represent height of signals (peaks, left scale), red squares indicate the potential at which the height of signals was detected (right scale). Spectrophotometric analysis of the ZnS QDs. (**I**) Spectrophotometric record of ZnS QDs within the range from 230 to 380 nm. (**J**) Detection of Zn(II) ions using spectrophotometric record of the coloured product of the reaction of ZnS QDs with 5-brom-PAPS with absorption maximum recorded at 550 nm.

Quantum dots (QDs) are fluorescent semiconductor nanocrystals in the size ranging from 1 to 20 nm. They have specific optical and electronic properties depending on the ratio of the surface area to volume and on the so-called “quantum confinement” phenomenon. As a developing semiconductor material star, ZnS nanoparticles (NPs) are material with low toxicity [57] and with a wide band gap [14]. They exhibit remarkable optical and electrical properties [58–60], which suggest that they may be particularly well-suited for manufacturing novel sensors [61,62]. ZnS quantum dots were studied by the use of microfluidic electrochemical analysis. Identically as in the previous compounds studied, the reduction potential of zinc in 20 μM ZnS QD was analysed (Figure 7A). The highest signal was observed under the use of 0.2 M acetate buffer at pH 5.5. The recorded signal increased on average by 11% with decreasing pH value (by 0.5 pH units) of the used buffer.

The movement from acidic (pH 3.5) to less acidic pH(pH 5.5) was accompanied by a significant shift of the potential to more negative values (Figure 7B) in the same way as that seen in Zn(phen)(his)Cl_2_ and Zn(his)Cl_2_. The increasing temperature (10–50 °C) led to the change in the height of signal and has almost no effect on the signal shift. The best results were recorded at a temperature of 20 °C, and −1.16 V applied potential (Figure 7C). Changes in potential were further investigated amperometrically within the range from −1.4 to −0.6 V. Applied potential of −1.2 V showed the highest signal (Figure 7D). The same results were observed in the case of zinc nitrate and Zn(his)Cl_2_ complex. Height of the signal increased up to the flow rate of 892 μL/mL, after that the recorded signal remained unchanged (Figure 7E). The most suitable time of accumulation was determined as 110 s, where the potential showed a moderate shift to more positive values with the increasing time of accumulation (Figure 7F). Calibration curve of Zn(II) ions using DPV is shown in Figure 7G. Dependence of concentration on the change in electrochemical signal was logarithmic, with the coefficient of determination R^2^ = 0.9802 (Figure 7G). Reduction signals of Zn(II) were also investigated by the use of HMDE. The dependence of concentration on the change in signal was linear with a coefficient of determination R^2^ = 0.9786 (Figure 7H).ZnS QDs were finally characterized by UV/VIS spectrophotometry the same way as the complexes and zinc nitrate studied previously. A moderate increase in absorbance at 313 nm was detected (Figure 7I). The presence of Zn(II) in 20 μM ZnS QD was proved spectrophotometrically in the same way as the complexes studied previously (Figure 7J).

### 3.5. Comparison of Effect of pH and Time of Accumulation on the Change in Potential

**Figure 8.**
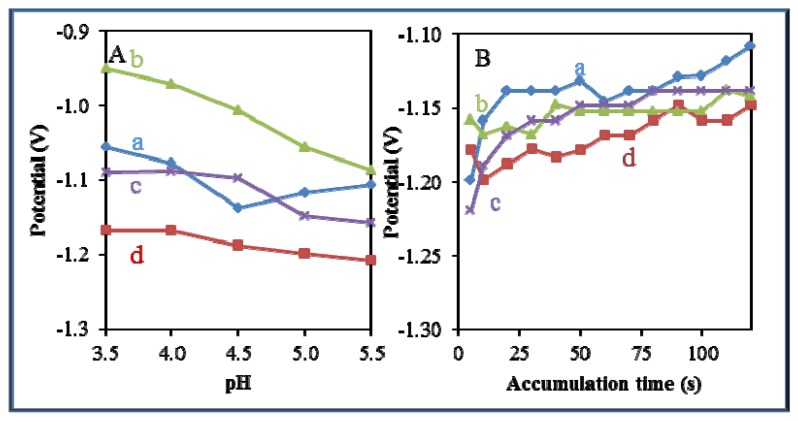
Dependence of height of signal on the change in pH value (3.5–5.5) and time of accumulation (5–120 s) of studied zinc-based compounds as (**A**) (a = zinc nitrate, b = Zn(phen)(his)Cl_2_, c = Zn(his)Cl_2_, and d = ZnS-QD), and (**B**) (a = zinc nitrate, b = Zn(phen)(his)Cl_2_, c = Zn(his)Cl_2_, and d = ZnS-QD).

A significant effect of pH and time of accumulation on the change in potential was observed in all studied zinc-based compounds and quantum dots. Changes in pH led to a shift of the electrochemical signal to more negative values (Figure 8A). The most significant effect of pH was recorded for Zn(phen)(his)Cl_2_ (Figure 8A-b). On the other hand, an adverse effect was observed in the case of the time of accumulation, where potential shifted from negative to more positive values with the increasing time of accumulation (Figure 8B). The most significant effect of the time of accumulation on the change in potential was recorded for zinc nitrate (Figure 8B-a) and Zn(his)Cl_2_, (Figure 8B-c).

### 3.6. Summary of the Characterization Results

Table 1 summarizes the optimal conditions for the electrochemical study of zinc complexes and ZnS QDs using microfluidic analysis. Investigated zinc compounds were studied at a potential of −1.2 V, with the exception of Zn(phen)(his)Cl_2_, which showed an electrochemical signal at the potential of −1 V. The most suitable conditions for the study of complexes were achieved using 0.2 M acetate buffer pH 5.5 with the exception of zinc nitrate, which was well detected in the same buffer, but at pH 4.5. The largest differences were observed in the temperature optimization of the detection of zinc complexes. The complex Zn(his)Cl_2_ showed the strongest electrochemical signal at 10 °C, whereas at the complex Zn(phen)(his)Cl_2_ the strongest signal was achieved at 50 °C. The optimal temperature for the electrochemical analysis of zinc nitrate was 40 °C and for the analysis of ZnS QD it was 20 °C. For all investigated samples it was observed that with higher flow the electrochemical signal was also increasing. The highest signals were obtained under the flow rate 1,300 μL/min. Due to the saving of samples the optimal flow rate was always chosen. The lowest flow rate was set for nitrate (135.5 μL/min), while the highest was for ZnS QDs (892 μL/min). For Zn(phen)(his)Cl_2_ and Zn(his)Cl_2_ the same flow rate (320 μL/min) was used. The longest accumulation time (100 s) was set for Zn(his)Cl_2_ and ZnS QD. The shortest accumulation time (80 s) was chosen for Zn(phen)(his)Cl_2_ and zinc nitrate (90 s).

**Figure 9.**
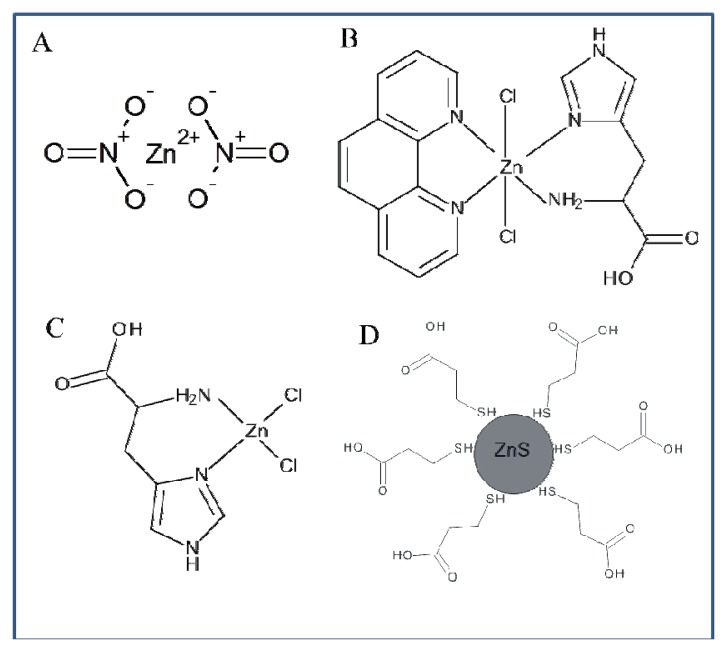
Structure of (**A**) Zinc nitrate and suggested structure of zinc complexes as (**B**) Zn(phen)(his)Cl_2_, (**C**) Zn(his)Cl_2_ and (**D**) ZnS QDs covered with 3-mercaptopropionic acid.

The whole work deals with the electrochemical study of zinc nitrate, zinc complexes and ZnS nanoparticles using a microfluidic flow system. The advantage of this system compared to HMDE is the possibility to miniaturize the entire system, which offers possibilities for other applications. The usability of HMDE is limited due to the high toxicity of mercury and small anodic range (limited by the oxidation of mercury). Another advantage of the applied method is the easy adjustability of different flow rates in order to achieve better electrochemical signals. When using HMDE these possibilities are not applicable. Printed electrodes can be modified by various substances in order to achieve better results in electrochemical analysis, but the options of HMDE in this respect are very limited. Flow systems containing printed electrodes are generally easier to use. HMDE method is more demanding on the instrumentation owing to the fact that falling of mercury drops may occur. Printed electrodes in combination with a microfluidic flow system can be an alternative to electrochemical methods using HMDE. The results presented in this study should serve as a basis for further investigation of zinc compounds. Based on the results obtained in this study we suggested the structure of all compounds of interest (Figure 9).

## 4. Conclusions

This study presents an electrochemical study of four zinc-based compounds by the use of flow cells and printed electrodes. The method was first optimized using zinc nitrate. We then monitored the electrochemical reduction signals of Zn(phen)(his)Cl_2_, Zn(his)Cl_2_, and ZnS QD nanoparticles. Complexes were further characterized by UV/VIS spectrophotometry, chromatography, and ESI-QTOF mass spectrometry. Complexes of zinc with 1,10-phenanthroline which is a universal chelating agent were described and the use of zinc nanoparticles has been growing [5,63–66]. Investigating these complexes is urgently required due to their biological activity. They are able to bind and cleave DNA. In addition, they present significant antimicrobial activity.

## Figures and Tables

**Table 1.** Summary table with the optimal conditions for microfluidic analysis.

	**Potential (V)**	**pH**	**Temperature (°C)**	**Flow Rate (μL/min)**	**Accumulation Time (s)**
Zinc nitrate	−1.2	4.5	40	133.5	90
Zn(phen)(his)Cl_2_	−1	5.5	50	640	80
Zn(his)Cl_2_	−1.2	5.5	10	320	120
ZnS QD	−1.2	5.5	20	892	110
